# Morphology of the lateral aspects of the human cortex: an informational explanation

**DOI:** 10.3389/fpsyg.2025.1525963

**Published:** 2025-08-22

**Authors:** Jeel Moya-Salazar, Hans Contreras-Pulache

**Affiliations:** Universidad Privada Norbert Wiener, Vicerrectorado de Investigación, Lima, Peru

**Keywords:** brain, information, neocortex, anatomy, brain lateralization, consciousness, society, paleocortex

## Abstract

This article presents a novel perspective on the structure and function of the human cortex, grounded in the Sociobiological Informational Theory (SIT). SIT offers a conceptual framework that integrates biological, psychological, and social dimensions of brain activity, challenging traditional anatomical and physiological models. Under this perspective, the neocortex is interpreted as the system of consciousness, while the paleocortex is associated with unconscious processes. This approach moves beyond classical models focused solely on anatomical structures, emphasizing instead the organized, dynamic nature of brain function as embedded in broader networks. The article argues that understanding the brain in isolation (from the nervous system, personal history, culture, and socioeconomic context) is insufficient for explaining human behavior and cognition. Consequently, SIT advocates for a rethinking of neurohistology, neuroanatomy, and neurophysiology, shifting from a static, disembodied view of the brain to a more integrated and coherent understanding of the living person—a new anatomy. This alternative interpretation of the human cortex, and of the body more broadly, opens avenues for interdisciplinary dialogue, encouraging exploration of the deep connections between consciousness, personality, and society.

## 1 Introduction

It is essential to begin by clarifying that the entire mass of neural tissue housed within the skull is known as the encephalon. This structure comprises several key macroscopic components, including the brainstem (which itself contains the medulla oblongata, pons, and midbrain), the cerebellum, the diencephalon (which includes the hypothalamus and thalamus), the telencephalic nuclei (such as the striatum and amygdala), and finally, the cortical telencephalon, commonly referred to as the cerebral cortex, cerebrum of human cortex.

It is important to emphasize that encephalon, brain, and human cortex are not synonymous; the human cortex represents only a part of the encephalon (which is equivalent to brain). This distinction becomes especially relevant in multilingual scientific communication. For example, the English term “brain” is often used to describe the entire encephalon but is frequently—and incorrectly—translated into Spanish as “cerebro” (that means human cortex). A clear example of this mistranslation is the phrase “Decade of the brain” (Jones and Mendell, 1999), commonly rendered in Spanish as “Década del cerebro,” when it would be more accurate to refer to it as “Década del encéfalo.” Such imprecision can lead to notable misunderstandings, particularly in scientific contexts where clarity and conceptual accuracy are critical.

With this clarification in mind, it becomes evident that an explanation of the human cortex is not equivalent to an explanation of the encephalon as a whole (Hernández-Yépez and Contreras Pulache, 2019). In this manuscript, we focus specifically on the morphology of the lateral aspects of the human cortex, interpreted through the lens of the Sociobiological Informational Theory (Moya-Salazar and Contreras-Pulache, 2023; Moya-Salazar and Contreras-Pulache, 2021; Contreras-Pulache et al., 2019a,b). We begin with a critical review of classical models of the human brain, proceed to introduce an informational perspective for understanding cortical structure and function, and conclude with a synthetic discussion that integrates both approaches.

## 2 The classic perspectives of the human cortex

The two cerebral hemispheres are not mirror images, neither at the macroscopic nor microscopic level, nor in terms of physiological or psychological function. Nonetheless, there is a broad morphological similarity between them (Bui and Das, 2023; Purves et al., 2001; Netter and Brass, 1994). Examining the lateral surfaces of the hemispheres reveals fissures, sulci, and gyri. Various explanations have been proposed to account for these anatomical features (Venkadesh and Van Horn, 2021; Batista-García-Ramó and Fernández-Verdecia, 2018).

The classical view maintains that, based on the distribution of fissures and reference landmarks, the human cortex can be divided into lobes. Traditionally, six lobes are recognized: frontal, parietal, temporal, occipital, insular, and limbic. Over time, however, brain segmentation has become more refined. In the early 20th century, Brodmann introduced a system of approximately 50 cortical areas based on cytoarchitectural features (Zilles, 2018). By the mid-20th century, further microstructural and clinical studies had expanded this classification to around 130 distinct regions (Hatta et al., 2002). More recently, advances in neuroimaging have enabled the identification of up to 180 functionally distinct areas in each hemisphere (Glaser et al., 2016). In less than two centuries, brain mapping has evolved from gross anatomical divisions into lobes to highly detailed functional parcellations (Wen et al., 2024; Pittella, 2024). This remains the dominant model taught in neuroscience education today.

Within this traditional model, the prevailing archetype for understanding the human cortex (and the human body in general) has been the study of the dead body. Since the establishment of modern anatomy over seven centuries ago, the central paradigm for explaining the human organism has been grounded in cadaveric dissection. An underlying belief seems to persist: that the better we understand the dead structure, the better we can understand the living person. For instance, the discovery of the neuron in the early 20th century marked a milestone in neuroscience and laid the groundwork for modern approaches. The contributions of Ramón y Cajal, followed by Brodmann’s cytoarchitectural mapping, significantly advanced our understanding of brain microstructure. These developments occurred alongside ongoing debates between localizationist and holistic perspectives. Yet, a fundamental factual assumption was overlooked: the subject under study was not a living neuron but a dead neuron. Thus, anatomy progressed from the macroscopic study of brain lobes to microscopic observations of neurons and cortical areas, yet always through the lens, or under the hegemony of the dead body.

In the early 1990s, when research paradigms started to emphasize the study of the living brain (and, in general, the living body: genome, connectome, microbiome, etc.). The emergence of what might be called a New Anatomy (focused on the structure and function of the living body rather than the structure of the dead body) began to challenge the classical cadaver-based model. Unlike traditional anatomy, which draws from the static and dissected body, this new paradigm centers on the living body, the living (and, obviously, psychic) brain.

On these lines, we propose that the traditional model is merely one way (a specific perspective) of understanding the human brain. This classical model, whether based on lobes, cytoarchitecture, or functional areas, is a product of cultural and historical developments over the past centuries. In contrast, the sociobiological informational explanation of the human brain offers an alternative framework. This approach is grounded in the Sociobiological Informational Theory (Contreras-Pulache et al., 2019a; Contreras-Pulache et al., 2018), developed by Pedro Ortiz Cabanillas in Peru, and published in 1994 (Ortiz, 1994).

In this context, we propose that the traditional model of the brain is but one possible framework (a culturally and historically contingent way). Whether it is based on lobes, cytoarchitecture, or functional regions, it reflects a specific epistemological era. In contrast, the Sociobiological Informational Theory (SIT), developed by Pedro Ortiz Cabanillas (1933–2011) in Peru, offers an alternative explanation of the human brain. First synthesized in its essential form in 1994 (Ortiz, 1994), and later expanded (Contreras-Pulache et al., 2018, 2019a), SIT provides a novel framework that moves beyond classical anatomical assumptions.

## 3 The informational explanation of the human cortex

The Sociobiological Informational Theory (SIT) is a comprehensive theoretical framework that offers a novel approach to understanding the development of living systems and human society. Proposed by Pedro Ortiz Cabanillas (1933–2011), SIT is grounded in dialectical materialism (Llontop, 2007) and can be epistemologically situated as a form of systems theory, a reinterpretation of Wiener’s cybernetics, and a general theory of information that encompasses Shannon’s specific quantitative model (Drack and Pouvreau, 2015; Wiener, 1948).

Ortiz redefines the classical notion of “information,” moving beyond the conventional understanding of it as mere messages, news or data. Instead, he conceptualizes information as “a material structure whose activity underpins the organization of all living beings, from bacteria to human society” (Ortiz, 2008). In this sense, information is not a communicative unit but an organizing principle of life (Ortiz-Cabanillas, 2017; Ortiz, 2010). In humans, this organization is based on social information, and the human brain is the organ capable of encoding and processing such symbolic, historical, and culturally embedded information (Contreras-Pulache et al., 2023; Contreras-Pulache et al., 2024).

From this informational perspective, the human cortex is not structured according to traditional divisions into lobes or into 50 or 180 functional areas. Rather, it is organized along two fundamental levels of psychic integration: the paleocortex and the neocortex. This conceptual shift requires a new understanding of brain-society integration (Ortiz, 2004), wherein the brain encodes not just sensory or motor signals but also knowledge, emotions, and values (forms of social information embedded in cultural, economic, and historical contexts).

Accordingly, the human brain plays as the substrate for psychic activity. The cerebral cortex is not merely a network of interconnected neurons, as seen under a microscope, but a functional structure capable of encoding psychic content (Ortiz, 2008). This perspective represents a transition from a strictly anatomical view to a psychobiological integrative model, opening new pathways to explore the link between consciousness and social meaning (Contreras-Pulache et al., 2020a, Contreras-Pulache et al., 2020b; Moya-Salazar and Contreras-Pulache, 2022).

Human beings, uniquely, can develop an internal symbolic reality. This capacity marks a rupture with animality. As Ortiz emphasizes, the human is not simply an economic, rational, or moral animal—it is no longer an animal at all, but a persona. This distinction is foundational in SIT: only humans, embedded in a symbolic and social world, attain true consciousness.

Thus, the human brain operates on two integrated levels. The paleocortex corresponds to the unconscious system, integrating all neural pathways from subcortical nuclei—both ascending and descending, sensory and motor. This definition of unconsciousness differs from traditional psychological or Freudian views; it is not about repressed content or lack of awareness, but about a mammalian psychic level (that of a human neonate or a primate). In contrast, the neocortex represents the conscious system, the seat of symbolic interaction and personal identity, structured by and within a sociohistorical context, and also structured as an experience from within.

Consciousness, in the informational framework, is not an ephemeral realization or a cognitive function, but a structural and functional property of the neocortex, or the neocortex itself. Specifically, it is grounded in regions such as the dorsolateral prefrontal cortex, anterior temporal cortex, orbitofrontal cortex, and parietotemporooccipital cortex. These areas integrate the nervous system and, through it, subsume the entire body. Thus, consciousness (neocortex) is defined both: as a structure and as an activity (Ortiz, 1994).

This conception diverges sharply from mainstream theories of consciousness in cognitive neuroscience and philosophy of mind. Authors such as Francis Crick and Christof Koch, for instance, define consciousness as the momentary awareness of mental content (Koch, 2004). By contrast, SIT defines consciousness as a continuous structural function, not a momentary event but the neurobiological embodiment of symbolic, intersubjective reality.

From this point of view, mammals and human neonates are unconscious, not because they lack momentary awareness, but because their neural activity is governed by the paleocortex. Even when an animal appears to “realize” something, it remains unconscious in the informational sense, as it lacks symbolic, cultural, and historical embeddedness. If animals had culture, economy, politics, and tradition, they would be conscious. The neocortex, in SIT, exists to biologically sustain that transcendence.

Hence, while a monkey may exhibit some form of self-awareness, its brain remains paleocortical in function. Without the symbolic structuring provided by society, it cannot generate true consciousness. Consciousness is not a precondition for society—it is a product of it. As such, it is not an inherent attribute of higher primates, but an emergent property of the human persona within society. This also means that a person cannot be truly unconscious. A person exists only insofar as consciousness exists. In cases such as severe dementia, the biological human being may persist, but the social identity—the person—disappears. A parallel case is depicted in François Truffaut’s film “The Wild Child,” where a child raised outside society fails to develop consciousness. Even if he exhibits awareness, it is not symbolic consciousness in the informational sense (Contreras-Pulache et al., 2019b). In short: being aware is not the same as being conscious, and being unconscious is not the same as lacking awareness.

**Figure 1:**
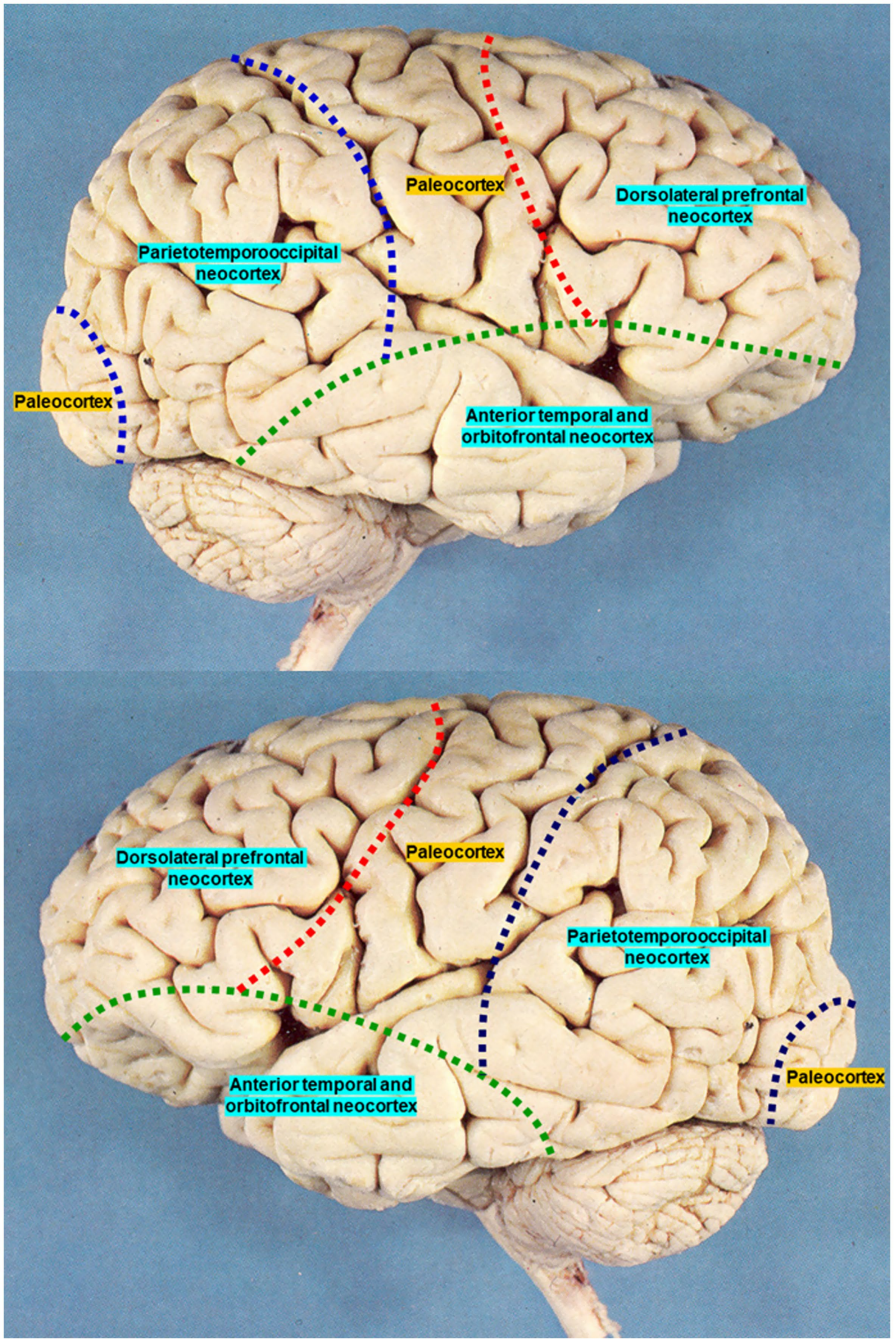
Both lateral sides of the human cortex. Dashed lines designed by Pedro Ortiz Cabanillas, based on the conceptual framework of Sociobiological Informational Theory, using two classical lateral views from his original teaching slides at Universidad Nacional Mayor de San Marcos in postgraduate neuroscience program (2009).

To explore this model in anatomical terms, we begin with Figure 1, which shows a lateral view of the human cortex, highlighting the informational distinction between the neocortex and paleocortex. The cortical distribution outlined with dashed lines was originally designed by Pedro Ortiz Cabanillas as part of his teaching at the Universidad Nacional Mayor de San Marcos around 2009, during a general psychobiology course in the postgraduate neuroscience program. This program is widely recognized as the leading school of neuroscience in Peru. Both cortical images presented here were taken directly from the original lecture slides used by Ortiz in his classes. The paleocortex includes primary cortical areas, while the neocortex comprises associative cortices. In humans, the neocortex is more extensive and, according to SIT, tripartite in structure.

Ortiz proposes that the neocortex functions as a unified system (the conscious activity), yet structurally, it displays a triune organization. This idea links his model with a long tradition—from Plato to Luria—that sought to explain the human psyche in tripartite terms (see Table 1). Ortiz, however, is unique in fully integrating this with the nervous system, the human body, and the structure of society. Thus, from an informational viewpoint, the person, society, and neocortex are all organized tripartitely.

**Table 1:** The triune view perspective.

Author	First component	Second component	Third component
Plato	Appetite	Reason	Will
Jackson	Lower level	Middle level	Higher level
Pavlov	Unconditioned reflexes	Conditioned reflexes	Second signal system
Freud	Id	Ego	Superego
MacLean	Reptilian brain	Mammalian brain	Primate brain
Luria	Unit for regulating tone, wakefulness, and mental states	Unit for receiving, analyzing, and storing information	Unit for programming, regulating, and verifying activity
Person (Ortiz)	Affective system	Cognitive system	Conative system
	Anxiety	Attention	Expectation
	Dispositions	Aptitudes	Attitudes
	(Temperament)	(Intellect)	(Character)
	(Behavior)	(Performance)	(Conduct)
Society (Ortiz)	Traditional	Cultural	Economic
	Religion	Philosophy	Science
Neocortex (Ortiz)	Anterior temporal and orbitofrontal neocortex	Parietotemporooccipital neocortex	Dorsolateral prefrontal neocortex

Data compiled from original lecture slides by Pedro Ortiz Cabanillas in Universidad Nacional Mayor de San Marcos (2009), as part of his teaching within the framework of Sociobiological Informational Theory.

To fully understand the human cortex, one must examine the lateral, medial, and basal views of both hemispheres. In this article, we focus on the lateral view, which is both the most familiar and most culturally recognized of human brain’s views.

According to SIT, the human brain consists of two integrated hemispheres, each operating at two psychic levels: the unconscious (limbic and heterotypic cortex) and the conscious (transition and association cortex). The definitions of paleocortex and neocortex used here are informational, not merely anatomical.

As previously discussed by Marín-Padilla (2011), the brain is a central evolutionary innovation in mammals. Informationally, we argue that the human cortex comprises two distinct levels: the paleocortical (unconscious) shared with mammals, and the neocortical (conscious), unique to humans who live in society (Moya-Salazar and Contreras-Pulache, 2021; Ortiz, 2004). Conscious psychic activity is thus structurally grounded in the neocortex (Ortiz, 1998, 2007, 2008).

Figure 1 illustrates this tripartite neocortical organization in both hemispheres. Importantly, although these regions differ between hemispheres, what matters most is their integration, not their differentiation. Hence, we propose the notion of interhemispheric complementation, a concept with potential quantum implications that we will explore in future work.

This new framework challenges classical neurohistology, neuroanatomy, and neurophysiology. We must rethink how nervous tissue is shaped by social information—be it cultural, traditional, or economic. Neuroanatomy must move beyond the cadaver; it must become a science of the living persona. Neurophysiology must evolve to address not only the activity of the nervous system but also its structure–activity relationship: structured activity and activated structure. In this sense, SIT proposes a truly new anatomy.

## 4 Conclusion

An alternative model of brain structure is proposed, based on a synthetic visual representation of the lateral aspects of both cerebral hemispheres. Rooted in the Sociobiological Informational Theory (SIT), this framework redefines brain function as structured activity—particularly of the cortex—rather than static anatomy. In this view, the neocortex encodes conscious memory and social information, forming the basis of consciousness, while the paleocortex operates as a system of unconscious memory—the informational unconscious.

This model is part of a broader theory linking brain, person, society, and universe. SIT moves beyond neurocentrism, integrating historical, cultural, and economic dimensions into neuroscience. It challenges reductionism and invites interdisciplinary dialogue with philosophy, psychology, and the social sciences.

Importantly, SIT is a Latin American theory, formulated in Spanish from the Global South. It offers fresh tools for understanding the human cortex as part of a living, culturally embedded person—moving beyond traditional post-mortem anatomy toward a dynamic, situated perspective.

## Data Availability

The original contributions presented in the study are included in the article/supplementary material, further inquiries can be directed to the corresponding author.
